# Antimuscarinic Toxicity Safely Managed with High-Dose Transdermal Rivastigmine: A Case Report

**DOI:** 10.5811/cpcem.49089

**Published:** 2026-01-16

**Authors:** C. James Watson, Emilie M. Burrill, William S. Jaffee

**Affiliations:** * Maine Medical Center, Department of Emergency Medicine, Portland, Maine; † Northern New England Poison Center, Portland, Maine; ‡ Tufts University School of Medicine, Boston, Massachusetts; § Maine Medical Center, Acute Care Pharmacy Services, Portland, Maine; ¶ Maine Medical Center, Adult Inpatient Medicine, Portland, Maine

**Keywords:** drug overdose, muscarinic antagonists, physostigmine, rivastigmine, case report

## Abstract

**Introduction:**

Antimuscarinic toxicity, which can cause delirium and unsafe behavior, may result from an adverse effect of prescribed medications or from non-medical substance use. Physostigmine shortages have prompted use of transdermal rivastigmine for management of antimuscarinic toxicity; however, symptom control is equivocal at standard dosing.

**Case Report:**

A patient with antimuscarinic toxicity was treated with physostigmine and transitioned to 26.6 milligrams/24 hours transdermal rivastigmine for sustained symptom control. He experienced no adverse effects and tolerated floor admission.

**Discussion:**

There is mechanistic plausibility supporting safe, sustained control of antimuscarinic toxicity with high-dose transdermal rivastigmine. Central distribution is more rapid than serum distribution and higher doses correlate with a shorter time to peak concentration.

## INTRODUCTION

Physostigmine is the gold standard antidote for antimuscarinic toxicity (AMT).^1^,^2^ In 2023, persistent shortages of US-manufactured physostigmine led the US Food and Drug Administration to authorize importation of a German alternative Anticholium—physostigmine salicylate (Dr. Franz Köhler Chemie GmbH, Bensheim, Germany).^3^,^4^ The imported physostigmine remains scarce, however, and the shortage is ongoing. To manage AMT without requiring critical care admission or physostigmine re-dosing, physicians are using other centrally acting acetylcholinesterase inhibitors for symptom management.^5^–^9^

Transdermal rivastigmine is one such option. It is formulated in 4.6 milligrams (mg) per 24 hours, 9.5 mg/24 hour, and 13.3 mg/24 hour patches with a maximum approved dose of 13.3 mg/24 hours for Alzheimer dementia and Parkinson disease.^10^ Existing literature on transdermal rivastigmine for AMT challenges its efficacy and time to clinical effect, particularly given the slower onset of the transdermal formulation as compared to oral rivastigmine. However, the literature reports the same dosing for Alzheimer dementia.^5^–^9^ To our knowledge there have been no prior published reports of higher dose transdermal rivastigmine for the management of AMT.

Patients with AMT are, by definition, experiencing direct muscarinic receptor antagonism. We hypothesize that this cohort requires transdermal rivastigmine in higher doses than used for Alzheimer dementia to achieve adequate symptom control. We argue that previous studies of transdermal rivastigmine for AMT were limited by insufficient dosing. We present a novel case of AMT requiring physostigmine for initial symptom control that was promptly, effectively, and safely maintained without critical care admission on high-dose 26.6 mg/24 hour transdermal rivastigmine.

## CASE REPORT

A 42-year-old, 63-kilogram man with polysubstance use disorder presented to the emergency department (ED) from an inpatient detoxification center for abnormal behavior. He had enrolled in the center one day prior for medically supervised management of withdrawal from fentanyl, freebase cocaine, and ethanol. Earlier on the day of ED presentation, he exhibited erratic behavior described as kicking walls, thrashing, and moaning. His behavior was presumed related to substance withdrawal, and he was treated at the detox center with 20 mg oral olanzapine, 50 mg oral hydroxyzine, 1,500 mg oral methocarbamol, and unspecified doses of oral gabapentin, trazodone, dicyclomine, and clonidine. He had worsening mentation, nonsensical speech, and frequent unsuccessful attempts to urinate on the floor, prompting his ED evaluation.

At ED triage (time 00:00), the following vital signs were recorded: heart rate, 98 beats per minute; blood pressure, 135/90 millimeters of mercury; respirations, 19 breaths per minute; temperature, 36.8 °Celsius; pulse oximetry, 97%; and capillary glucose, 137 mg per deciliter. Promptly after arrival he was evaluated by the ED resident and attending physician, who was also a consultant toxicologist with the regional poison center. The patient provided no history, and no additional information was available from the detox center. His exam was notable for restlessness, trying to climb over the gurney railing, grabbing at space, picking at his telemetry leads, and biting at his pulse oximeter. He vocalized with nonsensical, muffled, mumbling sounds. He had nonreactive 3-millimeter pupils bilaterally, flushed skin, dry axillae, tachycardia, and mild tachypnea. There was no clonus, and patellar reflexes were 1+ bilaterally. There was no tremor and no tongue fasciculations. He repeatedly grabbed at his genitals in unsuccessful attempts to urinate. Qualitative ultrasound demonstrated a large bladder volume. Laboratory results from 00:21 (Table) were reassuring against acute metabolic disturbance or common co-ingestion.

Clinical course is shown in the Figure. The patient received two benzodiazepine doses between 00:20 and 00:29 without effect. His presentation was consistent with AMT, and he received physostigmine for diagnostic and therapeutic purposes. Pre-administration, hyperactivity prevented an interpretable electrocardiogram (ECG); however, the QRS interval by telemetry was 80–100 milliseconds (ms). At 00:38, he received 2 mg intravenous (IV) physostigmine (*Köhler*) over five minutes. He subsequently had improved attention and ability to remain in bed. Otherwise, his speech, picking movements, and urinary retention were unchanged. At 01:01, he received another 2 mg IV physostigmine (*Köhler*) over five minutes followed shortly by abrupt, complete resolution of encephalopathy, hyperactive movements, and urinary retention. There were no findings consistent with alcohol or opioid withdrawal. He recalled being at the detox center previously, and that he had been given “a whole bunch of pills” from an unknown source and without additional detail. An ECG at 01:13 showed sinus tachycardia at 106 beats per minute, QRS of 99 ms, and QTc (Bazett) of 475 ms.

*CPC-EM Capsule*

What do we already know about this clinical entity?

*Transdermal rivastigmine (tdR) can be used to treat antimuscarinic toxicity (AMT) in the absence of physostigmine.*

What makes this presentation of disease reportable?

*Following high-dose tdR (26.6 mg/24hours) for AMT after initial physostigmine reversal, the patient’s symptoms did not recur while admitted on telemetry.*

What is the major learning point?

*High-dose tdR for AMT may be an effective means of maintaining symptom control. We recommend telemetry monitoring for those receiving high-dose tdR.*

How might this improve emergency medicine practice?

*High-dose tdR may improve AMT and reduce intensive care admissions in the era of physostigmine shortages.*

Anticipating AMT recrudescence and given limited physostigmine availability, transdermal rivastigmine was administered. Due to the presenting illness severity and the bedside physician’s experience with incomplete symptom control from 13.3 mg/24 hour transdermal rivastigmine, two 13.3 mg/24 hour transdermal rivastigmine patches were placed on the upper back at 01:36 (total 26.6 mg/24 hour). Appreciating the possible time to clinical effect, application was expedited. The patient was admitted to a telemetry hospitalist service, but he continued boarding in the ED. Given the novel use of high-dose rivastigmine, monitoring for muscarinic toxicity was discussed with the admitting hospitalist, and arrangements were made for the poison center to follow the case.

The patient developed no further AMT or muscarinic toxicity. At 13:20, both rivastigmine patches were removed. There was no concern for suicidal intent. He was discharged at 35:21 after receiving subcutaneous, extended-release buprenorphine. Serum testing performed at 16:08 resulted post-discharge. Hydroxyzine/cetirizine were detected at 27 nanograms per milliliter (ng/mL) and 220 ng/mL (5 ng/mL and 50 ng/mL reporting limits, respectively). Diphenhydramine was undetectable (50 ng/mL reporting limit). Olanzapine concentrations were not collected. The patient provided informed consent for the publication of this case.

## DISCUSSION

We present the case of an adult with AMT resolved with IV physostigmine, whose symptom control was maintained outside a critical care setting using high-dose transdermal rivastigmine without adverse effect. Acute AMT is classically managed with physostigmine, which has a short time to effect of < 10 minutes but is limited by a short duration of effect of one to two hours.^11^,^12^ The physostigmine shortage has resulted in substitution with more widely available, centrally acting acetylcholinesterase inhibitors including rivastigmine.^5^–^9^

Transdermal rivastigmine has benefits compared to the oral formulation: 1) easier administration in those intolerant of oral medications or enteric tubes; 2) no dependence on normal gastrointestinal absorption kinetics; and 3) once-daily dosing with more consistent tissue concentrations.^6^,^13^ However, two retrospective cohort studies found that transdermal rivastigmine was less effective than oral rivastigmine at controlling AMT. Greene found that among 22 AMT patients, those receiving transdermal rivastigmine alone (9.5–13.3 mg/24 hours) had a five-hour median time to symptom control compared to those also receiving oral rivastigmine (two hours).^8^ Similarly, Chiew et al found that among 50 AMT patients, 73% of those receiving transdermal rivastigmine (mode 9.5 mg/24 hours) required additional sedation compared to 32% of those receiving oral rivastigmine.^9^ Both investigators proposed that transdermal rivastigmine’s slower time to serum peak concentration (T_MAX_; 8–16 hours^10^) was contributory to its inferior effects.^8^,^9^

Our patient, however, transitioned from requiring multiple doses of physostigmine to high-dose transdermal rivastigmine without recurrent AMT. This may indicate that transdermal rivastigmine was clinically effective within one to two hours of being placed, as it is mechanistically plausible that higher doses facilitate more rapid, complete symptom control. Transdermal rivastigmine’s serum T_MAX_ shortens as the dose increases.^14^ Furthermore, rivastigmine’s rapid central distribution results in a cerebral spinal fluid (CSF) T_MAX_ of only 1.4–2.6 hours despite the lagging serum T_MAX_.^10^ Rivastigmine’s clinical effects in patients with Alzheimer dementia correlate directly with CSF acetylcholinesterase activity, which in turn correlates with rivastigmine dose.^15^ Therefore, we theorize that the higher transdermal rivastigmine dose contributed to more rapid CSF acetylcholinesterase inhibition and onset of clinical effect before the physostigmine was no longer effective.

Safety—that is, monitoring against muscarinic toxicity—was a primary consideration when implementing high-dose rivastigmine. While not requiring critical care, the patient underwent telemetry monitoring for the duration of his rivastigmine treatment, and atropine was immediately available. We were comfortable pursuing a high dose due to the following considerations: 1) the tolerability profile of transdermal rivastigmine in patients with Alzheimer dementia even at 17.4 mg/24 hour^10^; 2) the absence of documented adverse events attributed to transdermal rivastigmine in previous AMT cases^5^,^6^,^8^,^9^; 3) strong communication with a collaborative hospitalist team with oversight by the regional poison center; and 4) the toxicodynamic principle that developing muscarinic symptoms from higher rivastigmine doses would be unlikely given the clear muscarinic receptor antagonism our patient was already experiencing. While this patient demonstrated no muscarinic toxicity, we still recommend telemetry monitoring in future cases.

An unexpected benefit of the physostigmine shortage has been the development of treatment protocols requiring less-frequent dosing than is allowed by IV physostigmine, an issue previously raised by Dawson and Buckley.^12^ Physostigmine has a one to two hour duration of effect and is typically administered in either an ED or critical care setting given the historic risk of precipitous muscarinic toxicity or seizure.^12^ In the case presented here, transitioning to transdermal rivastigmine was viewed as protective against the need for additional physostigmine or critical care services; therefore, the patient was admitted to a telemetry floor. Without durable AMT control, the admitting team would have requested an intensive care unit admission. If physostigmine becomes broadly available again, physostigmine bolus followed by transdermal (or oral) rivastigmine maintenance should be considered for resource conservation.

This case report has limitations. First, generalizability is limited and causative conclusions cannot be made. Second, while it is possible that the patient would have had a similar course without transdermal rivastigmine or with a dose lower than 26.6 mg/24 hour, the need for two physostigmine doses for initial symptom control and the lack of any muscarinic effects with a high rivastigmine dose argue against this possibility. Third, while the patient’s examination and response to physostigmine clearly support AMT, he never mounted tachycardia to the degree generally expected with this toxidrome. This may be related to clonidine administered prior to transfer from the detox center. Fourth, there is no definitive causative agent for the patient’s symptoms. Hydroxyzine and cetirizine were detected late in the clinical course at inconclusive concentrations;^16^ it is unknown how these compounds contributed to the initial presentation or whether other antimuscarinic agents (olanzapine) were involved. The clinical toxidrome and its resolution with physostigmine, however, support this as a case of AMT.

## CONCLUSION

High-dose transdermal rivastigmine (26.6 mg/24 hour) may provide efficient, effective, and safe symptomatic control following physostigmine bolus in carefully selected patients. Those receiving high-dose transdermal rivastigmine require telemetry monitoring and prompt availability of atropine, but they may not need critical care admission. Larger cohort studies are indicated to identify the appropriate transdermal rivastigmine dose for this population.

## Figures and Tables

**Figure:**
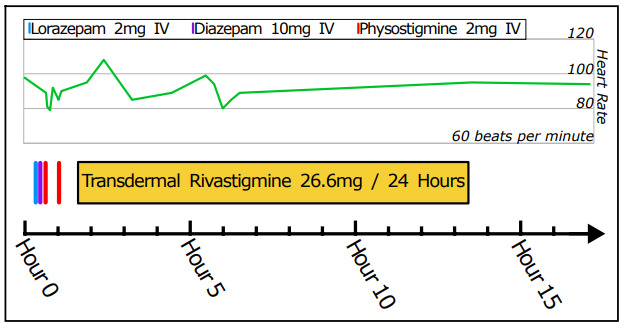
Clinical course: Shortly after arrival, the patient received a clinical diagnosis of antimuscarinic toxicity. He was treated with both lorazepam and diazepam with minimal effect and subsequently received two doses of IV physostigmine 2 mg and had complete resolution of his symptoms. Transdermal rivastigmine (26.6 mg/24 hour) was placed. The patient developed no recurrent antimuscarinic toxicity nor did he develop any muscarinic adverse effects from the rivastigmine; his heart rate remained in the normal range. The rivastigmine was discontinued, and the patient was discharged on hospital day two. *IV*, intravenous.

**Table 1:** Laboratory values on presentation in a patient suspected of having antimuscarinic toxicity.

Laboratory Test	Value (reference range)
Hemoglobin	14.6 g/dL (12.3–16.9)
Leukocytes	8.6 thousand/μL (3.6–11.8)
Platelets	385 thousand/μL (142–390)
Sodium	142 mEq/L (135–145)
Potassium	4.2 mEq/L (3.5–5.1)
Chloride	106 mEq/L (96–108)
Magnesium	2.3 mg/dL (1.6–2.6)
Carbon dioxide	23 mEq/L (21–30)
Anion gap	13 mEq/L (7–16)
Blood urea nitrogen	14 mg/dL (6–20)
Creatinine	0.87 mg/dL (0.5–1.3)
*Glucose	141 mg/dL (70–99)
Aspartate transferase	16 U/L (8–48)
Alanine transferase	17 U/L (7–55)
Bilirubin (Total)	< 0.2 mg/dL (< = 1.2)
Lipase	26 U/L (13–60)
Creatine kinase	93 U/L (39–308)
Lactate	1.1 mmol/L (0.5–2.0)
Acetaminophen	< 5 μg/mL
Salicylate	< 10 mg/L
Ethanol	< 10 mg/dL

* Abnormal value.

dL, deciliter; g, gram; L, liter; mEq, milliequivalent; mg: milligram; mmol: millimole; μg: microgram; μL: microliter; U: unit.
